# Automated Quantitative Assessment of Retinal Vascular Tortuosity in Patients with Sickle Cell Disease

**DOI:** 10.1016/j.xops.2024.100658

**Published:** 2024-11-22

**Authors:** Jimmy S. Chen, Fritz Gerald P. Kalaw, Eric D. Nudleman, Nathan L. Scott

**Affiliations:** 1 Viterbi Family Department of Ophthalmology, Shiley Eye Institute, University of California, San Diego, La Jolla, California; 2 UCSD Health Department of Biomedical Informatics, University of California San Diego, La Jolla, California

**Keywords:** Sickle cell disease, Sickle cell retinopathy, Vascular tortuosity, Computer-based image analysis, Artificial intelligence

## Abstract

**Objective:**

To quantitatively assess the retinal vascular tortuosity of patients with sickle cell disease (SCD) and retinopathy (SCR) using an automated deep learning (DL)-based pipeline.

**Design:**

Cross-sectional study.

**Subjects:**

Patients diagnosed with SCD and screened for SCR at an academic eye center between January 2015 and November 2022 were identified using electronic health records. Eyes of unaffected matched patients (i.e., no history of SCD, hypertension, diabetes mellitus, or retinal occlusive disorder) served as controls.

**Methods:**

For each patient, demographic data, sickle cell diagnosis, types and total number of sickle cell crises, SCD medications used, ocular and systemic comorbidities, and history of intraocular treatment were extracted. A previously published DL algorithm was used to calculate retinal microvascular tortuosity using ultrawidefield pseudocolor fundus imaging among patients with SCD vs. controls.

**Main Outcome Measures:**

Cumulative tortuosity index (CTI).

**Results:**

Overall, 64 patients (119 eyes) with SCD and 57 age- and race-matched controls (106 eyes) were included. The majority of the patients with SCD were females (65.6%) and of Black or African descent (78.1%), with an average age of 35.1 ± 20.1 years. The mean number of crises per patient was 3.4 ± 5.2, and the patients took 0.7 ± 0.9 medications. The mean CTI for eyes with SCD was higher than controls (1.06 ± vs. 1.03 ± 0.02, *P* < 0.001). On subgroup analysis, hemoglobin S, hemoglobin C, and HbS/beta-thalassemia variants had significantly higher CTIs compared with controls (1.07 vs. 1.03, *P* < 0.001), but not with sickle cell trait variant (1.04 vs. 1.03 control, *P* = .2). Univariable analysis showed a higher CTI in patients diagnosed with proliferative SCR, most significantly among those with sea-fan neovascularization (1.06 ± 0.02 vs. 1.04 ± 0.01, *P* < 0.001) and those with >3 sickle cell crises (1.07 ± 0.02 vs. 1.05 ± 0.02, *P* < 0.001).

**Conclusions:**

A DL-based metric of cumulative vascular tortuosity associates with and may be a potential biomarker for SCD and SCR disease severity.

**Financial Disclosures:**

Proprietary or commercial disclosure may be found in the Footnotes and Disclosures at the end of this article.

Sickle cell disease (SCD) is an inherited hemoglobinopathy characterized by abnormal erythrocytes prone to deforming, or “sickling,” under hypoxic conditions.^1^^,^^2^ Pathogenic variants of SCD include hemoglobin SS (HbSS), hemoglobin SC (HbSC), and hemoglobin S with Beta-thalassemia (HbS/Beta-Thal or HbS/Beta-Thalassemia), while carriers of one hemoglobin S gene, or those with sickle cell trait, may not demonstrate SCD. Normally, fetal hemoglobin (HbF, the dominant form of fetal hemoglobin composed of 2 a-globin and 2 g-globin subunits) is replaced by adult hemoglobin (HbA, composed of 2 a-globin and 2 b-globin subunits) at 6 to 12 months of age.^3^ In SCD, a b-globin unit is pathogenically replaced by an HbSS, a variant prone to sickling.^4^ Erythrocyte sickling results in vaso-occlusions systemically, which include acute pain crises, acute chest syndrome (ACS), pulmonary embolisms, and sickle cell retinopathy (SCR).^5^ Consequently, SCD is an important cause of morbidity and mortality in affected patients, resulting in 34 400 deaths directly attributable to SCD and associated with all-cause mortality of 376 000 deaths worldwide in 2021.^6^

Sickle cell retinopathy is an important cause of vision loss in patients with SCD. In North American Black patients, the incidence of sickle cell trait is 8%, while that of SCD is 0.4%.^7^ The Jamaican Cohort Study previously demonstrated that SCR prevalence was 43% in HbSC and 14% in HbSS by 20.5 years of age.^8^ Risk factors for developing SCR include older age, smoking, hematuria, and decreased hemoglobin F.^9^^,^^10^ Clinical manifestations of SCR can be further divided into nonproliferative SCR (NPSCR) and proliferative SCR (PSCR), characterized by retinal neovascularization and fibrovascular proliferation in response to ischemic retinopathy.^9^^,^^11^ Proliferative SCR is most commonly treated by anti-VEGF^12^ or laser photocoagulation.^13^ Vision-threatening complications of SCR include vitreous hemorrhage and tractional retinal detachment, which requires surgical intervention. Thus, annual screening for patients >10 years old is recommended for SCD patients for SCR.^14^

Although it is well-established that vascular tortuosity reflects retinal ischemia and correlates with disease severity for ischemic retinopathies such as retinal vein occlusions^15^ and retinopathy of prematurity,^16^^,^^17^ few studies have validated vascular tortuosity quantitatively with overall SCD and SCR severity.^18^, ^19^, ^20^ Current screening focuses on hallmarks of SCR, including peripheral arterial occlusion, sea fan neovascularization, and salmon-patch hemorrhages and does not incorporate vascular tortuosity. Although deep learning (DL)-based methods have previously been published for detecting sea fan neovascularization in SCR,^21^ prior work for quantifying vascular tortuosity has focused on semiautomated computer-based image analysis with scanning laser ophthalmoscopy^18^^,^^19^ and OCT angiography (OCTA)^20^ in context of SCR. However, both scanning laser ophthalmoscopy and OCTA are not routinely used in SCR screening, and the methodologies used in these studies are limited by manual labor and may not be scalable for big data analytics. Recent studies focused on developing DL-based pipelines for automated vessel segmentation have successfully established the utility of vascular tortuosity for disease severity in retinopathy of prematurity^22^ and oxygen-induced retinopathy^23^ in large data sets. There remains a gap in knowledge regarding how these DL-based tools could assist in quantifying vascular tortuosity as a potential biomarker for SCR disease severity.

To address this gap in knowledge, the purposes of our study were twofold as follows: (1) to validate DL-based methods for calculating vascular tortuosity from fundus photos of patients with SCR and (2) to assess the vascular tortuosity metric as a clinical biomarker for SCD and SCR severity.

## Methods

This study was approved by the institutional review board at the University of California, San Diego, and adheres to the Declarations of Helsinki. Due to the retrospective nature of this study, the need for informed consent was waived by the University of California, San Diego institutional review board.

### Cohort Selection

Patients were identified using structured queries of the University of California, San Diego electronic health record data warehouse (Epic SlicerDicer). Patients with a medical history or a diagnosis of “Sickle-cell disorders” (International Classification of Diseases-10-Clinical Modification: D57.∗) were identified. Data from unaffected patients (i.e., no history of SCD, hypertension, diabetes mellitus, or retinal occlusive disorder) were also extracted and served as controls and were age- and race-matched to the SCD cohort. The cohorts were then limited to those who visited the eye center between January 1, 2015, and November 5, 2022.

Medical records for all included patients were manually reviewed for demographic information (age, race, ethnicity, and gender). For all patients with SCD, the following information was also extracted: sickle cell diagnosis (i.e., HbSS, HbSC, HbS/Beta-Thalassemia, and sickle cell trait), types of crises prompting an emergency department visit or hospitalization (pain crisis, ACS, priapism, etc.), the number of known total lifetime crises requiring a visit to the hospital, medications used (i.e. hydroxyurea, crizanlizumab, etc.), ocular comorbidities (including SCR history), systemic comorbidities, and any history of treatment (VEGF injection, laser, or surgery) as well as their indications. If available, peripheral ultrawidefield pseudocolor photograph findings and OCT photographs of the macula were extracted from the electronic health record for each included eye as well.

### Image Processing for Calculation of Cumulative Tortuosity Index

**Figure 1:**
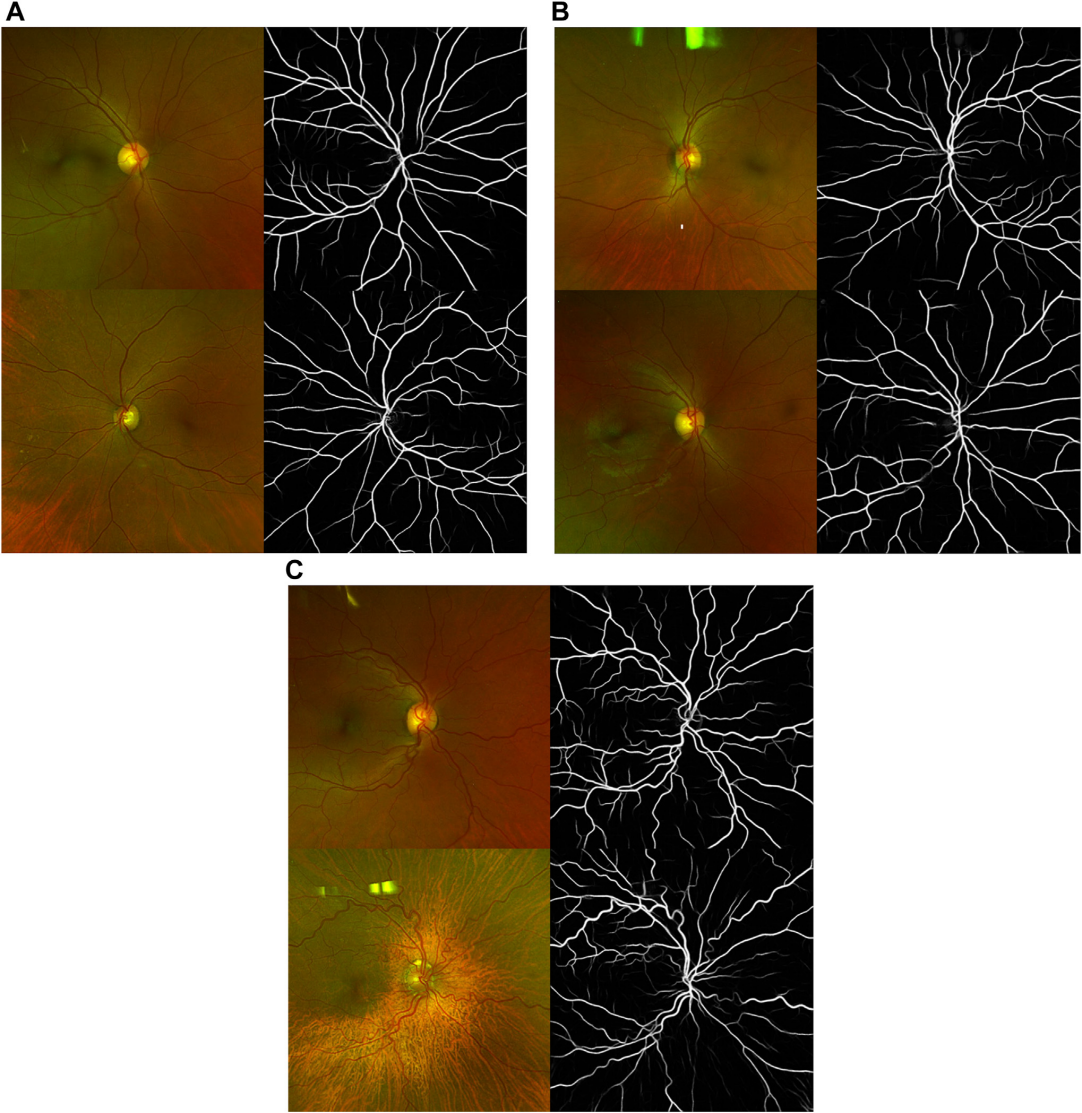
Cropped fundus photographs and vessel segmentations for unaffected controls (**A**) and sickle cell disease patients with nonproliferative sickle cell retinopathy (**B**) and sickle cell retinopathy (**C**).

Due to the retrospective nature of this study, the most recent ultrawidefield images of both eyes for each patient included in this study were extracted from our imaging Picture Archiving and Communicating System. These images were previously obtained as part of routine care using the Optos Ultra-Widefield Retinal Imaging Camera and uploaded per institutional protocol to our imaging Picture Archiving and Communicating System, Forum Viewer (Zeiss). Images were included if there was no distortion involving the posterior pole vasculature (i.e., excluded total retinal detachments with macular or peripapillary involvement). The included images were then centered on the optic nerve head and cropped to 400 × 400 pixels, thus excluding peripheral pathology. This cropping was performed to assess the response of posterior pole vascular tortuosity independent of peripheral disease and due to artifact from photography methods (i.e., eyelashes) and peripheral findings (i.e., salmon patches) that may be erroneously segmented from AutoMorph. Each image was then converted to 24-bit and input into AutoMorph, a DL-based pipeline for automated vessel segmentation from ultrawidefield fundus photographs.^24^ Examples of raw, cropped, and vessel-segmented images for representative control, NPSCR, and PSCR are shown in Figure 1. Each vessel segmentation was then resized to 512 × 512 pixels for input into iROP-Assist, a computer-based image analysis algorithm that calculates various vessel metrics such as vascular tortuosity.^22^ For this analysis, we chose to use the cumulative tortuosity index (CTI), as it has been previously validated for other diseases in prior studies.^23^^,^^25^^,^^26^

### Statistical Analysis

Data analysis was performed using R (version 4.0.5). Mean CTIs were calculated for all groups (control vs. SCD). Subgroup analyses for patients with SCD with and without SCR were performed for the following variables: medications used, number of crises (≥3 crises), and history of treatment for differences in CTI between subgroups. “Medications used” was defined in a binary manner: whether a patient with SCD used a drug intended for SCD treatment vs. not. History of PSCR vs. NPSCR vs. no SCR was also stratified into the following binary variables for analysis: PSCR vs. none, and any SCR vs. none. Due to the small sample sizes for HbS/Beta-Thal and sickle cell trait, further subanalysis by genotype was deferred. Imaging findings on Optos and OCT were also extracted in a binary manner (presence vs. absence) for univariable analysis. Shapiro–Wilk tests were used to assess normality. Two-tailed Student *t* tests for variables with normal distributions and Mann–Whitney *U* tests for variables with nonnormal distributions were used to assess statistical significance, with *P* ≤ 0.05 thresholded for significance. For univariable analysis of CTI vs. factors associated with SCD severity, Bonferroni correction was used to correct for multiple comparisons, ultimately yielding *P* ≤ 0.003 as the threshold for significance.

## Results

Overall, 106 eyes from 57 age- and race-matched patients without SCD and 119 eyes from 64 patients with SCD were included in this analysis. The mean age was 33.9 ± 20.5 vs. 35.1 ± 20.1 years for control patients and patients with SCD, respectively (*P* = 0.2). There were no statistically significant differences between race, ethnicity, and gender. Demographic data are provided in Table 1.

**Table 1:** Demographics of Controls vs. Sickle Cell Disease Patients

	Control	Sickle Cell Disease
Age (years, mean ± standard deviation)	33.9 ± 20.5	35.1 ± 20.1
Sex [n (%)]		
Female	39 (68.4%)	42 (65.6%)
Male	18 (31.6%)	21 (32.8%)
Did not disclose	0 (0.00%)	1 (1.6%)
Ethnicity [n (%)]		
Hispanic	2 (3.5%)	2 (3.0%)
Non-Hispanic	53 (93.0%)	60 (93.8%)
Multiracial	0 (0.0%)	1 (1.6%)
Unknown	2 (3.5%)	1 (1.6%)
Race [n (%)]		
White	5 (8.8%)	4 (6.3%)
Black	46 (80.7%)	50 (78.1%)
Other	5 (8.8%)	5 (7.8%)
Unknown	1 (1.7%)	5 (7.8%)
Total	57	64

Of the 64 SCD patients who underwent SCR screening, 28 (43.8%), 21 (32.8%), 6 (9.4%), and 9 (14.0%) had HbSS, HbSC, HbS/beta-Thal, and sickle cell trait, respectively. There were 10, 11, and 2 HbSS, HbSC, and HbS/beta-Thal patients with PSCR, respectively, and 7, 2, and 2 HbSS, HbSC, and HbS/beta-Thal patients with NPSCR, respectively. The mean number of crises per patient was 3.4 ± 5.2, for which the most common crises were pain (n = 31), ACS (n = 23), and deep venous thrombosis, or pulmonary embolism (n = 10). Patients with SCD were, on average, taking 0.7 ± 0.9 medications, which included hydroxyurea (n = 24), voxelotor (n = 12), crizanlizumab (n = 5), and glutamine (n = 3). The most common ocular comorbidities for patients with SCD were SCR (n = 34), glaucoma or glaucoma suspect (n = 9), retinal detachment (n = 5), dry eye syndrome (n = 5), and epiretinal membrane (n = 5). Patients were treated with laser (n = 17) and anti-VEGF drugs (n = 2) for proliferative retinopathy. Six patients with HbSS underwent surgery in this cohort: 5 for tractional retinal detachment and 1 for nonclearing vitreous hemorrhage. This data are shown in Table 2.

**Table 2:** Characteristics of Patients with Sickle Cell Disease, Including HbSS, HbSC, HbS/Beta-Thalassemia, and Sickle Cell Trait

	HbSS	HbSC	HbS/Beta-Thalassemia	Sickle Cell Trait	Overall
Number of crises per patient (mean ± standard deviation)	5.0 ± 6.4∗	1.7 ± 1.5∗	7.2 ± 6.7∗	0.0 ± 0.0	3.4 ± 5.2
Crises type (n)					
Pain	17	10	4	0	31
Acute chest syndrome	17	3	3	0	23
Avascular necrosis	3	1	2	0	6
Deep venous thrombosis or pulmonary embolism	4	3	3	0	10
Splenomegaly	3	1	0	0	4
Priapism	3	1	0	0	4
Dactylitis	2	1	0	0	3
Papillary necrosis	0	1	0	0	1
Immune thrombocytopenic purpura	0	0	1	0	1
Number of meds per patient (mean ± standard deviation)	1.0 ± 0.7∗	0.4 ± 1.0∗	1.3 ± 0.8∗	0.0 ± 0.0	0.7 ± 0.9
Medications used (n)					
Glutamine	1	2	0	0	3
Voxelotor	7	1	4	0	12
Crizanlizumab	1	3	1	0	5
Hydroxyurea	18	3	3	0	24
Most common ocular comorbidities (n)					
Sickle cell retinopathy	17	13	4	0	34
Retinal detachment	4	1	0	0	5
Dry eye syndrome	0	1	1	3	5
Glaucoma suspect/glaucoma	1	1	3	4	9
Epiretinal membrane	1	4	0	0	5
Retinal holes/tears	1	1	0	1	3
Age-related macular degeneration	1	0	1	0	2
Strabismus	1	0	1	0	2
Sickle cell retinopathy diagnosis (n)					
None	11	8	2	9	30
Nonproliferative	7	2	2	0	11
Proliferative	10	11	2	0	23
Treatment (n)					
Laser	6	9	2	0	17
Anti-VEGF	2	0	0	0	2
Surgery	6	0	0	0	6
Total [n (%)]	26 (40.6%)	20 (31.3%)	6 (9.4%)	9 (18.8%)	64

HbSC = hemoglobin SC; HBSS = hemoglobin SS.

∗ *P* < 0.05 when compared to patients with sickle cell trait.

### CTIs

**Figure 2:**
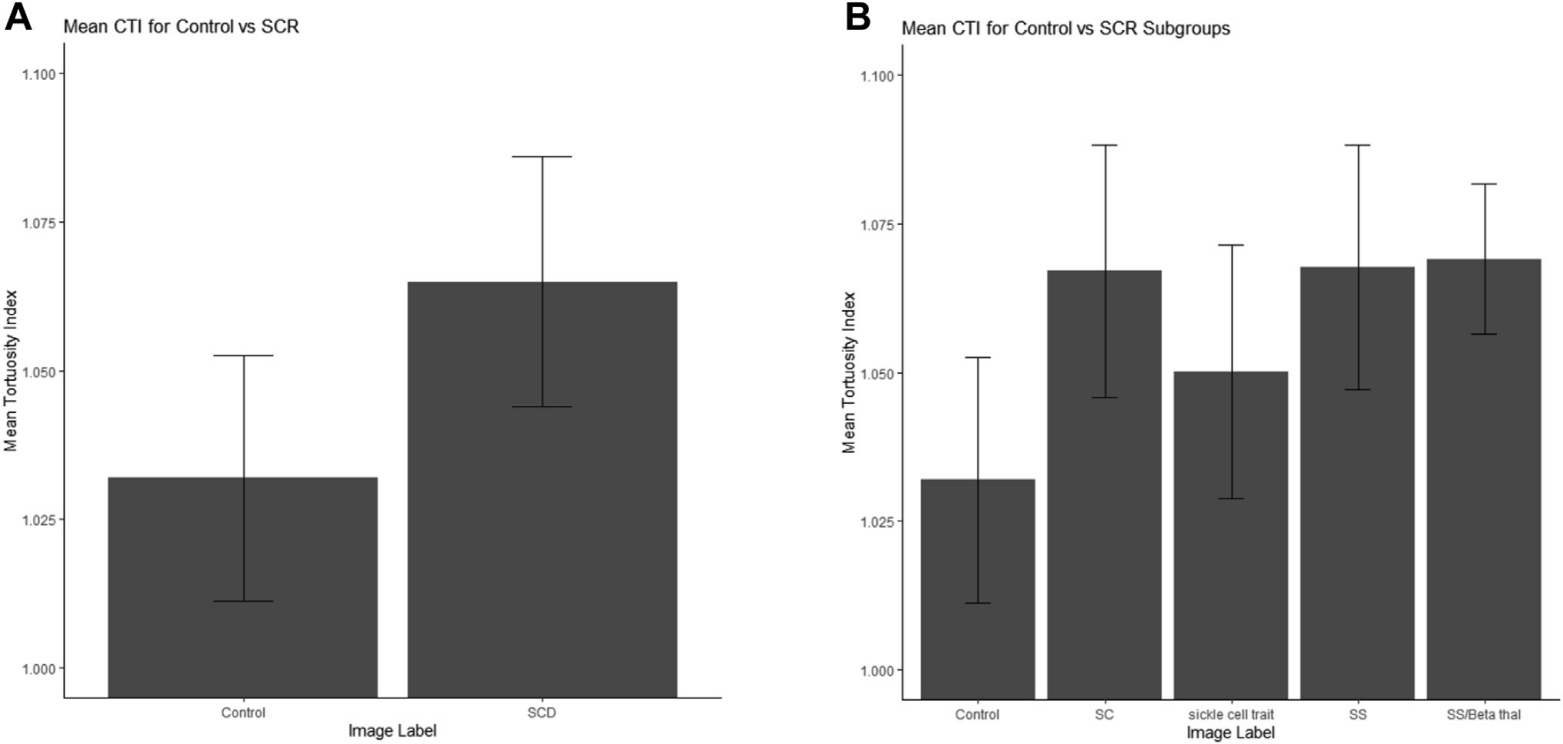
CTIs for control vs. sickle cell disease patients (**A**) and subgroup analysis by HbSS, HbSC, HbS/beta-Thal, and sickle cell trait patients (**B**). CTI = cumulative tortuosity index; HBsC = hemoglobin C; HbSS = hemoglobin S; HbS/beta-Thal, hemoglobin S with beta-thalassemia; SCD = sickle cell disease; SCR = sickle cell retinopathy.

The mean CTI for control eyes vs. eyes with SCD was 1.03 ± 0.02 vs. 1.06 ± 0.02, respectively (*P* < 0.01) (Figure 2A). On subgroup analysis between control vs. patients with various genotypes of SCD, the CTI for HbSS, HbSC, and HbS/beta-Thal patients were 1.07 ± 0.02, 1.07 ± 0.02, and 1.07 ± 0.01, respectively, when compared with control (*P* < 0.001). There was no statistically significant difference between HbSS vs. HbSc, HbSc vs. beta-thalassemia, and HbS/beta-Thal (*P* = 0.56, 0.81, and 0.40). There was also no statistically significant difference between CTIs for sickle cell trait (CTI: 1.04 ± 0.02) and control (*P* = 0.2) (Figure 2B).

### Analysis of Sickle Cell Patient Characteristics

A subgroup analysis was performed for patients with SCD and with and without SCR. Several features from the electronic health record chart review were analyzed in a univariate analysis to identify if higher vascular tortuosity was associated with clinical factors consistent with SCD severity. Although higher CTI was not found to be associated with the presence of SCR (*P* = 0.5), salmon patch (*P* = 0.10), or sunburst lesions (*P* = 0.51) on pseudocolor imaging, it was significantly associated with sea fan neovascularization, PSCR, and laser photocoagulation (*P* < 0.001 respectively). Similarly, CTI was found to be associated with a history of prior treatment (laser, anti-VEGF, or surgery, *P* < 0.001) and retinal detachment (*P* < 0.001). Cumulative tortuosity index was additionally found to be statistically associated with a number of hospitalizations for sickle cell crises (*P* < 0.001) and a number of SCD medications (*P* = 0.002). These data are shown in Table 3.

**Table 3:** Univariable Analysis of Cumulative Tortuosity Index Associations with Other Predictors of Sickle Cell Retinopathy and Sickle Cell Disease Severity

Variable	Mean Cumulative Tortuosity Index (Mean ± Standard Deviation)		*P* Value∗
	Variable Present	Variable Absent	
Ultrawidefield imaging findings			
Sunburst	1.07 ± 0.02	1.06 ± 0.02	0.51
Salmon patch	1.06 ± 0.01	1.06 ± 0.03	0.10
Sea-fan neovascularization	1.06 ± 0.02	1.04 ± 0.01	**<0.001**
Laser photocoagulation	1.06 ± 0.01	1.04 ± 0.02	**<0.001**
Fibrous proliferation	1.07 ± 0.02	1.04 ± 0.02	0.04
Vessel whitening	1.07 ± 0.02	1.04 ± 0.03	0.30
History of…			
Sickle cell retinopathy	1.06 ± 0.02	1.06 ± 0.02	0.54
Proliferative sickle cell retinopathy	1.07 ± 0.02	1.06 ± 0.02	**<0.001**
Retinal detachment	1.06 ± 0.01	1.04 ± 0.02	**<0.001**
Prior treatment	1.06 ± 0.02	1.04 ± 0.01	**<0.001**
Glaucoma	1.05 ± 0.03	1.06 ± 0.02	0.70
Retinal holes/tears	1.06 ± 0.02	1.05 ± 0.02	0.49
Diabetic retinopathy	1.06 ± 0.02	1.05 ± 0.03	0.58
Age-related degeneration	1.05 ± 0.03	1.04 ± 0.03	0.68
Sickle cell disease			
Admission for >3 crises	1.07 ± 0.02	1.05 ± 0.02	**<0.001**
Use of sickle cell disease medications	1.07 ± 0.03	1.04 ± 0.01	**0.002**

Bold = statistically significant ≤ 0.002.

∗ Due to repeated comparisons, Bonferroni correction was performed, with *P* < 0.003 as the threshold for statistical significance.

## Discussion

In this study, we used previously published DL-based methods to quantify vascular tortuosity in SCR. The cohort included in our study had demographic characteristics consistent with prior work studying SCR, with female patients of Black or African descent >30 years of age being the majority of the subjects. To further elucidate the impact of SCD on retinal vasculature, we compared patients with SCD with demographically matched controls with no concomitant diagnosis of SCD or systemic comorbidities that may cause intravascular compromise (i.e., hypertension, diabetes mellitus, and a prior history of retinal occlusive disease). Our study has 3 key findings: (1) patients with SCD have significant quantitative retinal vascular tortuosity compared with a control cohort, (2) CTI appears to be an independent biomarker of retinal vascular changes independent of SCR presence but may correlate with SCR severity, and (3) CTI is also associated with SCD disease severity, including a higher number of crises.

The first key finding is that patients with SCD have significant quantitative retinal vascular tortuosity compared with controls. In the present study, we demonstrate that applying DL-based algorithms trained on vessel segmentation and vascular tortuosity as a pipeline can be used for unprocessed pseudocolor fundus images centered along the optic nerve head region from patients with SCD and controls. This provides a potential methodology for quantitative and reproducible measurement of vascular tortuosity, as the exact definition of retinal vascular tortuosity is still unclear,^27^ and clinical assessment of vascular tortuosity remains subjective and highly variable to date.^28^ In the subjective sense, it can be construed as a vessel that is longer than a straight vessel over the same distance^29^ or a vessel that is dilated following a serpentine path.^30^ Quantitatively, retinal vascular tortuosity has been arduously experimented with in several retinal pathologies, and in patients with SCR, retinal vascular tortuosity has been investigated using an image processing algorithm primarily using OCTA.^25^^,^^31^, ^32^, ^33^, ^34^, ^35^ Several automated systems can currently classify tortuosity by assessing retinal vascular curvature.^22^^,^^24^^,^^29^ We utilized ultrawidefield pseudocolor imaging as it is used in routine clinical practice due to the ease of operation compared with OCTA, which is longer to perform and highly dependent on patient cooperation. Overall, patients with SCD were noted to have a higher tortuosity index than matched controls, consistent with its expected ischemic and vaso-occlusive pathophysiologic course. These findings have also been found in similar ischemic retinopathies including diabetic retinopathy,^35^ retinopathy of prematurity,^16^ oxygen-induced retinopathy,^23^^,^^26^ and retinal vein occlusions.^15^^,^^36^ However, the utility of vascular tortuosity in clinical assessment of these retinopathies remains unknown and limited by current metrics of vascular tortuosity.^28^

The second key finding in the present study is the significant retinal vascular tortuosity among patients with SCD diagnosed with SCR. More specifically, we found that patients with HbSS, HbSC, and HbS/beta-Thal demonstrated more severe vascular tortuosity compared with patients with sickle cell traits. Because ophthalmic manifestations of SCD can affect both the anterior and posterior segments of the eye,^11^^,^^37^ with retinopathy being the most common and the major cause of vision loss,^38^ screening with annual dilated fundus examinations starting at the age of 10 years is recommended.^14^ Interestingly, although prior work has demonstrated more severe PSCR in patients with HbSC and HbS/beta-Thal in context of retinal thickness,^39^ these studies did not evaluate vascular tortuosity as part of disease severity. Our study found similar findings, with HbSC and HbS/beta-Thal demonstrating the highest severity of CTI, although there was no statistically significant difference between vascular severity between HbSS, HbSC, and HbS/beta-Thal. Although this may be in part due to the higher propensity for vaso-occlusive events to occur in HbSS,^11^ larger studies for subgroup analyses between SCR severity may be needed to demonstrate significant differences. Furthermore, because the images utilized in our study were cropped to the central 400 × 400 pixels, only vessels in the posterior pole were included. In other words, no peripheral pathology characteristic of SCR (i.e., sea-fan neovascularization, salmon patches) was included. Therefore, CTI may be useful as a novel metric for SCR severity independent or in the absence of other known fundus findings, especially since CTI was not significantly associated with sunburst lesions and salmon patches. Further studies are needed to quantitatively determine if the degree of tortuosity increases with the progression of disease.

The third key finding is that CTI is also associated with SCD disease severity, including a higher number of crises. Although vaso-occlusion of peripheral vasculature from sickled erythrocytes initiates the process of organ damage,^40^ SCR in isolation does not often present with acute vision changes such as pain crises, deep venous thrombosis, or ACS, unless vitreous hemorrhage and retinal detachment occurs. Therefore, screening for SCR may give a window into a patient’s systemic status, a growing area of interest in ophthalmology especially in cardiovascular and Alzheimer disease.^41^, ^42^, ^43^ Additionally, early diagnosis of SCR at risk for developing peripheral findings, potentially by CTI, may prompt a hematologist or primary care provider to elevate care, which may have important implications for potentially preventing life-threatening complications of SCR. In our study, we demonstrated that higher CTI was associated with more tortuous retinal microvasculature in patients experiencing >3 crises and the use of medications for SCD. Prior work has also demonstrated associations between SCR and systemic factors such as the history of smoking, older age, higher hemoglobin and white blood cell counts, and hematuria.^10^ Because the associations found in our studies and other work do not establish a causal relationship between these features and systemic disease, more work prospectively will be needed to evaluate how risk factor modification alters the course of SCD both systemically and intraocularly.

The present study is limited by the relatively small number of pseudocolor imaging data from a single institution. Furthermore, the results may not be generalizable as the different SCD variants obtained in our cohort were not equally distributed. It is possible that there are different phenotypic expressions of the different variants in terms of retinal microvascular tortuosity. In addition, this is a retrospective study with limited fundus imaging data. A longitudinal analysis can provide valuable information regarding changes in CTI pre- and posttreatment or in patients converting from disease-free retina to NPSCR or PSCR.

In conclusion, using a pipeline of previously published DL algorithms, we demonstrate that retinal vascular tortuosity may serve as a potential biomarker of SCD and SCR severity. These data may offer a quantitative metric to follow in patients with SCD and SCR clinically and improve the quality of care delivered to these vulnerable patients. Future work may focus on assessing the utility of CTI as a biomarker in assessing disease severity, response to treatment, and disease chronicity for SCR and other ischemic retinal and vascular retinal diseases.
